# Paraneoplastic cerebellar degeneration associated with lymphoepithelial carcinoma of the tonsil

**DOI:** 10.1186/1471-2377-13-147

**Published:** 2013-10-17

**Authors:** Christian Henke, Johannes Rieger, Sylvia Hartmann, Marcus Middendorp, Helmuth Steinmetz, Ulf Ziemann

**Affiliations:** 1 Department of Neurology, Goethe University Frankfurt, Theodor-Stern-Kai 7, 60590 Frankfurt, Germany; 2 Institute of Neurooncology, Goethe University Frankfurt, Senckenberganlage 31, 60325 Frankfurtm, Germany; 3 Institute of Pathology, Goethe University Frankfurt, Senckenberganlage 31, 60325 Frankfurtm, Germany; 4 Department of Nuclear Medicine, Goethe University Frankfurt, Senckenberganlage 31, 60325 Frankfurtm, Germany; 5 Department of Neurology and Stroke, Hertie Institute for Clinical Brain Research, Eberhard-Karls-University Tübingen, 72076 Tübingen, Germany

**Keywords:** Lymphoepithelioma, Paraneoplastic syndrome, PET, Subacute cerebellar degeneration, Tonsil

## Abstract

**Background:**

Paraneoplastic cerebellar degeneration (PCD) is a classical tumor-associated, immune-mediated disease typically associated with gynecological malignancies, small-cell lung-cancer or lymphoma.

**Case presentation:**

Here we present the case of a 38-year old male with an over 12 months rapidly progressive cerebellar syndrome. Extensive diagnostic workup revealed selective hypermetabolism of the right tonsil in whole-body PET. Histological examination after tonsillectomy demonstrated a lymphoepithelial carcinoma of the tonsil and the tongue base strongly suggesting a paraneoplastic cause of the cerebellar syndrome. To the best of our knowledge this is the first case of an association of a lymphoepithelial carcinoma, a rare pharyngeal tumor, with PCD.

**Conclusions:**

In cases of classical paraneoplastic syndromes an extensive search for neoplasms should be performed including whole-body PET to detect tumors early in the course of the disease.

## Background

Paraneoplastic cerebellar degeneration (PCD) is a classical paraneoplastic syndrome (PNS) of the CNS [1] often associated with onconeural antibodies leading to a T-cell mediated destruction of Purkinje cells in the cerebellum [2]. Typically, PCD occurs in patients with ovarian cancer, breast-cancer, small-cell lung-cancer (SCLC) or Hodgkin’s lymphoma [2]. Nonetheless, PCD can be associated with other malignancies and can occur up to 5 years before detection of the primary tumor. Here, we describe a patient who developed subacute cerebellar degeneration which was classified as a paraneoplastic syndrome upon histological diagnosis of a lymphoepithelial carcinoma of the tonsil and tongue base. To the best of our knowledge, this is the first published case of PCD associated with a lymphoepithelial carcinoma.

## Case presentation

A 38-year old, previously healthy man presented on his first admission with a 5-day history of dizziness, slurring of speech and disturbance of gait and balance. There was a family history of multiple sclerosis (paternal grandfather and a paternal cousin) but not of any other cerebellar disease. The patient smoked 30 cigarettes per day for the last 25 years. Neurological examination revealed dysarthria, trunk and gait ataxia and exaggerated tendon reflexes of the upper and lower limbs. Spontaneous nystagmus was absent and pursuit eye movements were smooth. Routine hematology was normal but the cerebrospinal fluid (CSF) showed elevated cell count (407 lymphocytes/μl) and protein (1 g/l) while glucose and lactate were normal. CSF cytology provided no evidence of malignant cells but oligoclonal IgG bands were detected in the CSF. HSV- and VZV-PCRs were negative. Cerebellitis caused by an unknown infectious agent was suspected and a polyvalent intravenous therapy with aciclovir, ceftriaxone and ampicilline was initiated which halted disease progression. Cerebral MRI was unremarkable. During the next months he underwent two neurorehabilitation treatments without relevant efficacy on the residual symptoms, but 10 months after disease onset he rapidly deteriorated.

On admission this time he reported severe gait and trunk unsteadiness, difficulties in writing, massive slurring of speech and problems of swallowing. On physical examination he showed a substantial weight loss of 15 kg over the last 12 months but no enlargement of lymph nodes. Neurological examination revealed a cerebellar syndrome with an exaggerated horizontal nystagmus during lateral gaze and a scanning dysarthria. Fixation suppression of the vestibulo-ocular reflex was abolished. There was no muscle weakness or sensory loss and his limb reflexes were normal but plantar responses were bilaterally extensor. He showed severe dysmetria of all four limbs and trunk and gait ataxia. Walking without aid was impossible.

**Figure 1:**
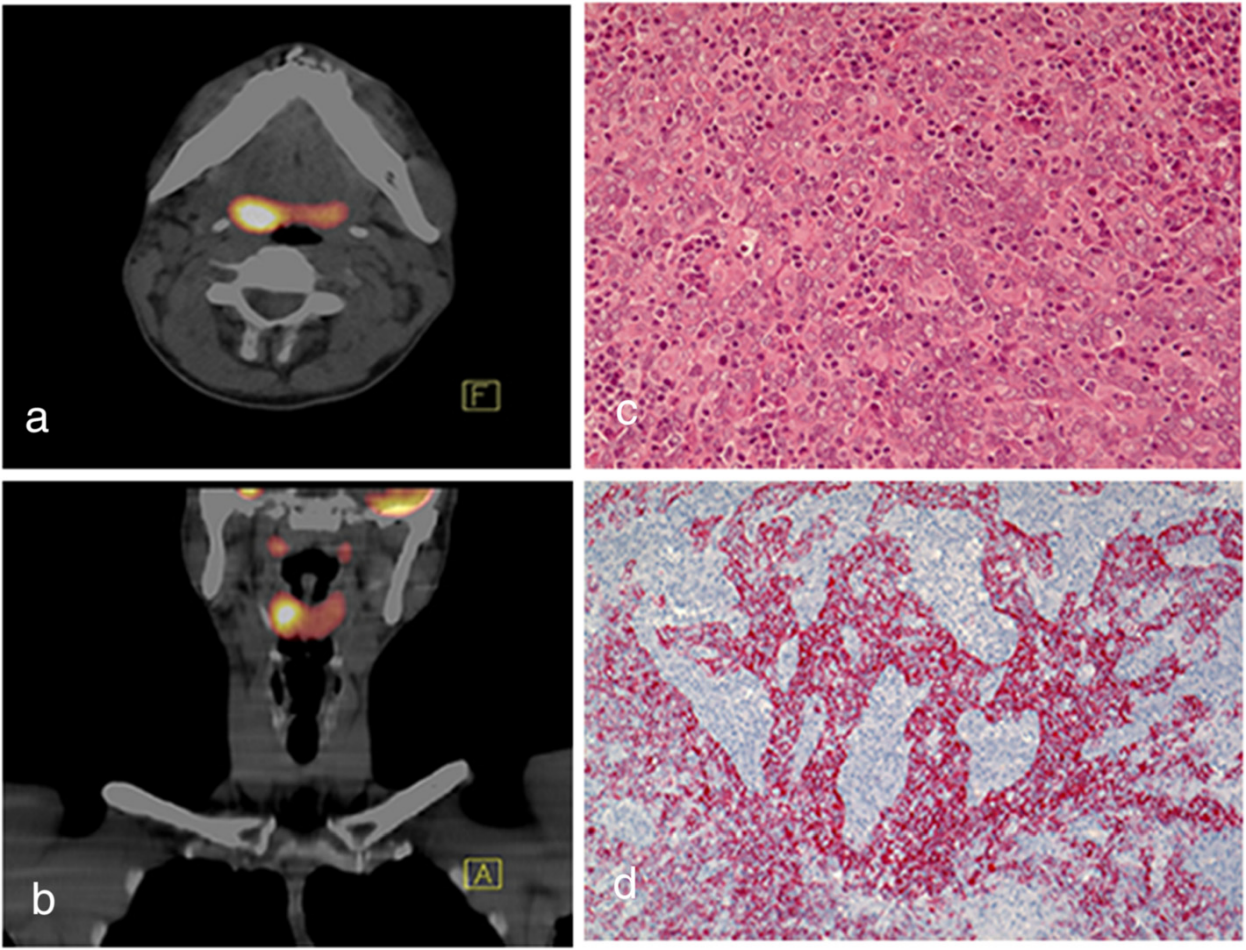
**Results of the radiological and histopathological investigation of the tonsils. a** and **b:** Whole-body PET/CT with [18F]-fluorodeoxyglucose (FDG) for tumor imaging according to the European Association of Nuclear Medicine (EANM) procedure guideline. Thick fused multi-planar reformatted images of the oropharynx in axial **(a)** and coronal **(b)** projection. Both images reveal an increased FDG uptake in the right tonsillar region suspicious for malignant tumor. **c and d**: Histopathology of the tonsil. HE hematoxylin-eosin [9] staining in 200 times- magnification **(c)** displaying sheets of atypical epithelial cells with enlarged nuclei and vesicular chromatin, intermingled by small lymphocytes and plasma cells. Pancytokeratin immunostaining **(d)** highlights the sheets of atypical epithelial cells (in red).

Routine biochemistry and hematology were again normal. Cranial MRI now showed marked cerebellar atrophy. The CSF contained 18 leukocytes/μl, 0.85 g/l protein, oligoclonal IgG bands, and normal glucose and lactate concentrations. CSF cytology was again normal. Infectiological screening of the CSF (HSV-1 and -2, VZV, CMV, EBV, HIV, treponema pallidum, brucella, borrelia) was negative. Further biochemical examination showed normal vitamins B12 and E and very-long-chain-fatty acids. Anti-gliadin-antibodies were negative. Squamous cell carcinoma-antigen (SCC) was mildly elevated (2.7 U/l, reference < 1.5) while NSE, PSA, CYFRA 21–1, CEA and electrophoresis were normal. Classical onconeural antibodies (anti-Hu, -Tr, -Yo, -Ma, -Ta, amphiphysin, CV2/CRMP5) were not detected via immunofluorescence test (IFT). MRI of the entire spinal axis did not show any metastases and search for a pulmonary or abdominal neoplasm in CT-scans of the chest and abdomen was also unremarkable. A whole-body PET/CT-scan revealed a mild hypermetabolism of the left thyroid lobe and the right tonsil and neighboring tongue-base (Figure 1a,b). Sonological and histological examination of the thyroid did not detect any malignancy. Inspection of the tonsils revealed no signs of a neoplasm except for an asymmetry with a larger right tonsil. Histological examination after tonsillectomy revealed a lymphoepithelial carcinoma of the lower tonsil and tongue base with low*-*grade differentiation (Figure 1c,d). The mitosis marker Ki67 was present in 40% of tumor cells. Immunohistochemical workup revealed numerous CD3+ T- and CD20+ B-lymphocytes with positivity for bcl2. Staining was negative for CD23, CD30, CD34, CD138, and the macrophage marker KiM1P. Additional resection of the neighboring tongue ground including neck dissection detected no further neoplastic tissue. Accordingly, the interdisciplinary tumor board did not recommend radio- or chemotherapy. In the course of the next eighteen months the patient underwent further intensive neurorehabilitation treatment leading to a stable cerebellar dysfunction with only slight progression of dysarthria which was treated every three months with steroid pulses (i.v. methylprednisolone 1 g/day for 5 consecutive days).

## Discussion and conclusions

This is the first published case of a lymphoepithelial carcinoma associated with PCD. Classification as a classical paraneoplastic syndrome and detection of a malignant tumor within twelve months after the onset of cerebellar symptoms establishes the diagnosis of a definite PNS [1]. Despite missing onconeural antibodies, the substantially slowed disease progression after tumor resection and the initial responsiveness to steroids largely exclude other differential diagnoses. The pathogenesis in our case as in most other PCD cases [2] is most likely a T-cell mediated cytotoxic autoimmune response against Purkinje cells [2].

Besides the typical tumor entities, i.e. ovarian cancer, breast cancer, lung cancer (mostly SCLC, but also adenocarcinomas) [2] a wide range of other tumors have been reported in association with PCD in individual cases (see Table 1, Additional file 1).

**Table 1:** List of PCD cases in PubMed not associated with gynecological, breast, or lung cancer, or lymphoma (state: December 2012; search key words: “paraneoplastic cerebellar degeneration”), f: female; m: male)

**Organ**	**Tumor**	**Sex**	**Age**	**Reference**
Bone marrow	multiple myeloma	f	57	Akpinar et al.
Thymus	thymic carcinoma	m	55	Bataller et al.
	thymic germinoma	m	55	Sola-Valls et al.
Systemic	Langerhans cell histiocytosis	3m, 2f	3,4,4,9,21	Goldberg-Stern et al.
Colon	Adenocarcinoma	f	55	Tsukamoto et al.
Stomach	gastric adenocarcinoma	m	73	Meglic et al.
	gastric carcinoid	m	58	Balducci et al.
Esophagus	Adenocarcinoma	m	57	Debes et al.
		m		Xia et al.
		m	55	Sutton et al.
Pancreas	small-cell carcinoma	m	52	Salmerón-Ato et al.
Kidney	renal cell carcinoma	m	66	Hens et al.
		f	64	Ammar et al.
Bladder	transitional cell carcinoma	f	64	Greenlee et al.
Testis	testicular seminoma	m	68	Kaluza et al.
	testicular germ cell tumor	m	47	van Warrenburg et al.
Prostate	Adenocarcinoma	m	68	Greenlee et al.
		m	79	Matschke J et al.
Skin	vaginal melanoma	f	52	Hauspy et al.
Nervous system	olfactory neuroepithelioma	m	65	Maeda et al.

(For references given in Table 1, please see Additional file 1).

The data in Table 1 reveal atypically localized malignancies in many different body regions belonging mostly to either the hematological/immunological system (lymphomas, Langerhans’ cell histiocytosis, thymus carcinoma), the gastro-intestinal system (esophagus, gastric, colon or pancreas carcinoma) or the urogenital system (kidney, bladder, testis or prostate cancer).

In our case histological examination demonstrated a lymphoepithelial carcinoma, which is a rare tumor entity, typically localized in the nasopharynx region. Due to the histological association with lymphoid tissue, tumor tissue can arise from all pharyngeal regions where lymphatic tissue is regularly located i.e. the tonsil, the hypopharynx, the tongue base and the paranasal sinuses [3-5]. Cases of lymphoepithelioma-like carcinomas of the salivary glands are also reported [6]. Lymphoepitheliomas are poorly differentiated tumors which contain tumor cells of epithelial origin in association with a diffuse infiltration of lymphocytes, plasma cells or eosinophils, which has led to coining to the term “lympho-epithelioma”. In some cases, also squamous cell carcinoma cells can be detected. The elevated tumor marker SCC in our case raises the possibility of a potential squamous cell-portion. No antibodies associated with lymphoma (anti-Tr) or SCLC (anti-Hu, -CV2, -Ri, -Ma2, -amphiphysin) were detected so that the origin of the antigenic structure that resulted in PCD remains unclear. We have not performed immunohistochemistry on rodent brain tissue sections with the patient’s CSF to search for new reactivities, which can be considered a limitation of this report.

There are only very few reports about paraneoplastic syndromes in association with lymphoepithelioma-like carcinomas of various other organs (polymyositis, nephrotic syndrome, erythema elevatum diutinum) [7-9] but onconeural antibodies have not been described in these cases. Classical neurological PNS have not yet been reported in association with this tumor entity so that this is the first case report of PCD associated with a lymphoepithelial carcinoma.

Due to the localized state of the tumor in our patient complete surgical removal of the carcinoma was feasible without additional radio- or chemotherapy. Regarding the pathophysiological concept of PNS the tumor resection is the most important part of the therapy to reduce the amount of antigenic tumor cells. In addition, immunomodulatory treatment with intravenous immunoglobulins occasionally shows efficacy in early stages of the disease [10].

Our case report highlights the usefulness of an extended tumor search - including a whole-body PET/CT scan - when a paraneoplastic syndrome is suspected [11]. A whole-body PET/CT scan provides superior sensitivity to detect small or atypically localized neoplasms, a precondition to initiate causal therapy by surgical removal.

## Consent

The patient gave his written and informed consent for this case report to be published.

## Competing interests

The authors declare that they have no competing interests.

## Authors’ contributions

CH, UZ, HS and JR contributed in drafting, writing and revising the article, MM and SH contributed in writing and revising the radiological and neuropathological discussion. All authors read and approved the final manuscript.

## Pre-publication history

The pre-publication history for this paper can be accessed here:

http://www.biomedcentral.com/1471-2377/13/147/prepub

## Supplementary Material

Additional file 1 **References of Table** 1**.** Click here for file
