# Supplementary material for: Paraneoplastic cerebellar degeneration associated with lymphoepithelial carcinoma of the tonsil
Source: BMC Neurol. 2013 Oct 17;13:147. doi: 10.1186/1471-2377-13-147 (PMC4016266; doi:10.1186/1471-2377-13-147)
Supplement: Additional file 1 — References of Table 1. [file 1471-2377-13-147-S1.docx]

**Supplementary material:**

References of table 1:

| AKPINAR S, BERK O, KARACA L. Paraneoplastic cerebellar degeneration associated with multiple myeloma - 3.4 years follow up*.* J Neurooncology 1990; **9**(1): 63-8 |
| --- |
| BATALLER L, VALERO C, DIAZ R, FROUFE A, GARCIA-ZARZA A, RIBALTA T, et al. Cerebellar ataxia associated with neuroendocrine thymic carcinoma and GAD antibodies*.* J Neurol Neurosurg Psych 2009; **80**(6): 696-7 |
| SOLA-VALLS N, GABA L, MUÑOZ E, MELLADO B, RIBALTA T, SAIZ A, et al. Paraneoplastic cerebellar degeneration associated with thymic germinoma. J Neurol Sci 2012; **320**(1-2): 153-5  GOLDBERG-STERN H, WEITZ R, ZAIZOV R, GORNISH M, GADOTH N. Progressive spinocerebellar degeneration "plus" associated with Langerhans cell histiocytosis: a new paraneoplastic syndrome? J Neurol Neurosurg Psych 1995; **58**(2): 180-3 |
| TSUKAMOTO T, MOCHIZUKI R, MOCHIZUKI H, NOGUCHI M, KAYAMA H, HIWATASHI M, et al. Paraneoplastic cerebellar degeneration and limbic encephalitis in a patient with adenocarcinoma of the colon. J Neurol Neurosurg Psych 1993; **56**(6): 713-6 |
| MEGLIC B, GRAUS F, GRAD A. Anti-Yo associated paraneoplastic cerebellar degeneration in a man with gastric adenocarcinoma. J Neurol Sci 2001; **185**(2): 135-8 |
| BALDUCCI G, FRONTONI M, BOCCHETTI T, ANGELINI D, DI GIACOMO G, ZIPARO V. Malignant gastric carcinoid and paraneoplastic cerebellar degeneration*.* Eur J Surg 1999; **165**(12): 1193-7 |
| DEBES JD, LAGARDE SM, HULSENBOOM E, SILLEVIS SMITT PA, TEN KATE FJ, SULTER GA, et al. Anti-Yo associated paraneoplastic cerebellar degeneration in a man with adenocarcinoma of the gastroesophageal junction*.* Dig Surg 2007; **24**(5): 395-7 |
| XIA K, SALTZMAN JR, CARR-LOCKE DL. Anti-Yo antibody-mediated paraneoplastic cerebellar degeneration in a man with esophageal adenocarcinoma*.* Md Gen Med 2003; **5**(3): 18 |
| SUTTON IJ, FURSDON DAVIS CJ, ESIRI MM, HUGHES S, AMYES ER, VINCENT A. Anti-Yo antibodies and cerebellar degeneration in a man with adenocarcinoma of the esophagus*.* Ann Neurol 2001; **49**(2): 253-7 |
| SALMERON-ATO P, MEDRANO V, MORALES ORTIZ A A, MARTINEZ-GARCIA FA, VILLAVERDE-GONZALEZ R, BAS A, et al. Paraneoplastic cerebellar degeneration as initial presentation of a pancreatic small-cell carcinoma. Rev Neurol 2002; **35**(12): 12-15 |
| HENS MJ, CAMACHO B, MAESTRE A, GONZALEZ V, VILLEGAS I, MAGAÑA M. Renal cell carcinoma presenting as paraneoplastic neurological dysfunction*.* Actas Urol Esp 2008; **32**(6): 645-8 |
| AMMAR H, BROWN SH, MALANI A, SHETH HK, SOLLARS EG, ZHOU SX, et al. A case of paraneoplastic cerebellar ataxia secondary to renal cell carcinoma*.* South Med J 2008; **101**(5): 556-7 |
| GREENLEE JE, DALMAU J, LYONS T, CLAWSON S, SMITH RH, PIRCH HR. Association of anti-Yo (type I) antibody with paraneoplastic cerebellar degeneration in the setting of transitional cell carcinoma of the bladder: detection of Yo-antigen in tumor tissue and fall in antibody titers following tumor removal*.* Ann Neurol 1999; **45**(6): 805-9 |
| KALUZA J, SLOWINSKI J, BUJNY T,GROCHALA M. Paraneoplastic syndrome simulating encephalitis in the course of testicular seminoma*.* Folia Neuropathol 1997; **35**(1): 24-8 |
| VAN DE WARRENBURG BP, RODRIGUEZ-JUSTO M, VELLA NR, FREEMAN A, BHATIA KP, QUINN NP. Paraneoplastic cerebellar ataxia due to burnt-out testicular germ cell tumour? Eur Neurol 2007; **57**(3): 178-81 |
| GREENLEE JE, CLAWSON SA, HILL KE, DECHET CB, CARLSON NG. Antineuronal antibodies in paraneoplastic cerebellar degeneration associated with adenocarcinoma of the prostate. J Neurol Sci 2010; Apr 15, **291**(1-2): 74-8 |
| MATSCHKE J, KROMMINGA A, ERBERSDOBLER A, LAMSZUS K, ANDERS S, KÖFÜNCÜ E. Paraneoplastic cerebellar degeneration and anti-Yo antibodies in a man with prostatic adenocarcinoma. J Neurol Neurosurg Psych 2003; **78**(7): 775-7 |
| HAUSPY J, NEVIN A, HARLEY I, MASON W, QUIRT I, GHAZARIAN D, et al. Paraneoplastic syndrome in vaginal melanoma: a case report and review of the literature. Int J Gynaecol Cancer 2007; **17**(5): 1159-63 |
| MAEDA K, SASAKI T, MURATA Y, KANASAKI M, TERASHIMA T, KAWAI H, et al. Paraneoplastic cerebellar degeneration in olfactory neuroepithelioma*.* J Neurol Neurosurg Psych 2006; **77**(1): 123-4 |
